# Localization in Structured Environments with UWB Devices without Acceleration Measurements, and Velocity Estimation Using a Kalman–Bucy Filter

**DOI:** 10.3390/s22166308

**Published:** 2022-08-22

**Authors:** Francesco Alonge, Pasquale Cusumano, Filippo D’Ippolito, Giovanni Garraffa, Patrizia Livreri, Antonino Sferlazza

**Affiliations:** 1 Department of Engineering, University of Palermo, Viale delle Scienze Ed. 10, 90128 Palermo, Italy; 2 Faculty of Engineering and Architecture, University of Enna KORE, 94100 Enna, Italy

**Keywords:** range-based localization without accelerometers, UWB, localization, velocity estimation, Kalman–Bucy filter

## Abstract

In this work, a novel scheme for velocity and position estimation in a UWB range-based localization system is proposed. The suggested estimation strategy allows to overcome two main problems typically encountered in the localization systems. The first one is that it can be suitable for use in environments where the GPS signal is not present or where it might fail. The second one is that no accelerometer measurements are needed for the localization task. Moreover, to deal with the velocity estimation problem, a suitable Kalman–Bucy filter is designed and it is compared, experimentally, with a particle filter by showing the features of the two algorithms in order to be used in a localization context. Additionally, further experimental tests are carried out on a suitable developed test setup in order to confirm the goodness of the proposed approach.

## 1. Introduction

Real-time localization of mobile objects and people has gained increasing interest and popularity because of the growing demand of location-based services such as in smart industries, warehouse management, healthcare industry, transportation and logistics, defense, mobile robot guidance, and autonomous drive and flight. For these reasons, the localization estimation problem is a challenge in the academic communities and industrial sectors, especially for those involved in autonomous flight applications design [1,2,3] where the controller, in order to implement real-time state-feedback control laws, has to know the position, linear and angular speed of the object, and its attitude. Since the position and velocity vectors, estimated by means of integrations of the accelerations measured by IMU, suffer uncertainties and disturbances introduced by the sensor itself, the aim of this paper is the estimation of the position of a moving point *P* and that of the velocity without acceleration measurements. In outdoor environments, where geostationary satellites are in LOS (line-of-sight) condition, the GPS signal is used to obtain information about position and velocity of a mobile object (see, for example, [4,5,6,7,8,9]), together with a data fusion system [10] which is able to handle the different nature and frequency of IMU measures and GPS signals. In other contexts, where the GPS signal becomes unreliable, for example, in indoor environments [11,12] or outdoor scenarios such as forests or urban canyons [13], it is necessary to use alternative sensor devices and methods to face the localization problem [14,15,16,17]. A common way to approach this kind of problem is to perform the navigation task in a structured environment where it is possible to take information about the distance between the moving object and some fixed reference points. This can be achieved, for example, by installing some UWB radio transceivers both on the mobile device and in correspondence of the fixed reference points, which are called anchors. The localization method is based on the measurement of the distances between the onboard module and the anchors, and makes use of a suitable algorithm to extract the position of the center of gravity of the moving object [18,19,20]. Starting from n≥3 distance measurements and applying a trilateration algorithm [21,22,23,24], it is possible to obtain an estimation of the position vector. With regards to the implementation of the trilateration algorithm, in order to calculate the best approximation of the mobile object position, some recursive procedures have been explored and proposed in the literature, generally based on linear and nonlinear least-square technique [25,26], or the use of Cayley–Menger determinant [27,28]. Other approaches based on Kalman filtering have also been investigated. For example, in [29], a Kalman filter based on data fusion algorithm was designed to improve the localization performance in an indoor scenario. In [30], a multi-sensor fusion algorithm for underwater vehicle localization was presented, making use of neural network and obtaining better navigation and localization results with respect to the classic implementation based on the error-state Kalman filter. In [31], a deep learning framework with multiple long short-term memory modules combined with an extended Kalman filter was proposed to predict the increment of the vehicle position in a localization task during GPS outages.

This paper represents an extension of the work presented in [32], where a localization scheme derived from the parallel robot context and based on the estimation of the direct kinematics is illustrated. In particular, assuming the gravity center of the mobile object as the end effector of a parallel robot and its distances from *n* anchors as the prismatic links, the estimation of the position vector is obtained by means of inversion of the known inverse kinematics. The distances from the anchors are measured at low frequency using the UWB radio transceivers DWM1000 integrated circuit (IC) from Decawave manufacturer [33]. The estimator consists of a closed-loop control scheme based on a PI action driven by the error between the measured distance vector and the estimated one, and affected by the distance vector derivative acting as a disturbance. Since this disturbance is calculated by means of IMU integration and a projection through the online-computed inverse kinematics, it is affected by errors due to drift caused by IMU biases. In order to cope with this effect, high values of the estimator gains have to be employed, as suggested in [34].

Since this solution appears to be unsatisfactory, in this paper, the estimator presented in [32] is modified, replacing the derivative of the distance vector with an estimation of this derivative carried out by means of an exact first-order Levant’s differentiator [35]. In this work, it is shown that it is possible to eliminate the accelerometer and all problems connected to it, such as systematic errors given by imprecise scaling factors, axes misalignments, non-uniform cross-axis sensitivities, non-zero biases, and temperature dependence. Moreover, from the theoretical point of view, it is possible to ensure the convergence to zero of the position estimation error in the case with three anchors, while if more than three anchors are employed, the convergence can be proved only locally. Finally, with the aim of also estimating the velocity of the mobile object, a Kalman–Bucy filter, driven by the estimated position, is introduced. It is important to highlight that the Kalman–Bucy filter input variables are computed by the proposed localization algorithm and, consequently, that they do not present drift problems caused by the double integration of the accelerometer measurement.

Experimental results show the effectiveness of the proposed localization scheme. In particular, it is shown that it is possible to approximate the first derivatives of the distances by using a suitable developed differentiator. This leads to a slightly noisier estimate, but allows to eliminate the accelerometer. Moreover, the Kalman–Bucy filter is compared with the particle filter, and it is shown that the best results are obtained using the differentiator and the Kalman–Bucy filter for velocity estimation, both in term of amplitude and variance of the estimation error.

The structure of the paper is as follows. In Section 2, the localization scheme for the position estimation without accelerometer measurements is illustrated by differentiating the case with three anchors (Section 2.3) and with more than three anchors (Section 2.4). The experimental results for this scheme are given in Section 2.5. In Section 3, we discuss three different schemes to estimate the velocity, also showing the experimental results and giving a comparison among them in Section 3.3. Finally, conclusions are given in Section 4. Additionally, the experimental setup implemented to prove the effectiveness of the proposed architectures is described in Appendix A.

## 2. Localization without Accelerometers

A trilateration algorithm, in both indoor and outdoor environments, allows to extract the information about the coordinates of a point *P* in a chosen reference frame by measuring the distances of *P* from some fixed devices whose coordinates $A_{i},i=1,\cdots ,n$ are a priori known. Since the aim of this paper is to propose a method for the localization of a point of a mobile moving object in indoor environments, where the GPS signals do not arrive, the electronic devices chosen to perform range-based measurements are ultra-wideband radio transceivers (see Section 1). The proposed method is based on the kinematics inversion algorithm highlighted in the parallel robotics field. In this context, differently from the one for serial robots, the structure of the inverse kinematics in a closed form is known, so it is necessary to determine the direct kinematics. Due to the nonlinear nature of the problem, this computation does not exist in a closed form. As already stated, the principal objective is that of performing the localization algorithm without any knowledge of accelerometer signals.

The inverse kinematics structure describing the relationship between the range measures $q_{i}$ and the coordinates p is given by the following equation:

(1) $$q=K^{-1}\left(p\right)$$

where the generic component of $K^{-1}\left(p\right)$, $q_{i}$, is given by:

(2) $$q_{i}\;=\;\sqrt{{\left(x_{i}\;-\;p_{x}\right)}^{2}+{\left(y_{i}\;-\;p_{y}\right)}^{2}+{\left(z_{i}\;-\;p_{z}\right)}^{2}}\;\;\forall i\in S,$$

where $x_{i}$, $y_{i}$, and $z_{i}$ denote, respectively, the components of the position of the *i*th anchor $A_{i}$ referred to the reference frame; $p_{x}$, $p_{y}$, and $p_{z}$ denote the coordinates of the mobile point *P*; $q_{i}$ are the distance between *P* and the *i*th anchor; and S={1,2,⋯n} denotes the discrete set of integer numbers representing the anchors. In the case under study, n≥3. In (1), the components of q and p represent, respectively, the prismatic link lengths and the coordinates of the reference point *P* of the parallel robot end-effector. If these coordinates ($p_{x}$, $p_{y}$, and $p_{z}$) are known, it is possible to obtain q by means of (2). However, the aim of this paper is to estimate the components of *P* by measuring the distances between *P* itself and the $A_{i},i=1,\cdots ,n$ anchors, and then the relationship to be used is the following one:

(3) p=K(q)

where K is not known and has to be determined.

In [22], it is shown that by using three anchors located on the ground plane, it is possible to obtain a closed-form solution of the direct kinematics problem with an uncertainty on the sign of the component along the *z*-axis. This uncertainty can be eliminated if the motion of the mobile object is restricted to one side of the plane on which the three anchors lie.

### 2.1. Determination of the Direct Kinematics for n≥3 Anchors

When more than three anchors are used, Equation (1) consists of *n* equations and three unknown quantities that are the coordinates of *P*. In this case, the problem is solved by means of the left pseudo-inverse algorithm. More precisely, deriving with respect to the time Equation (1), the following can be obtained:

(4) $$\dot{q}=J_{K^{-1}}\left(p\right)\dot{p},$$

where $J_{K^{-1}}\left(p\right)\in R^{n\times 3}$ is the Jacobian matrix of the vectorial function $K^{-1}\left(p\right)$, given by:

(5) $$J_{K^{-1}}\left(p\right)=\frac{\partial K^{-1}\left(p\right)}{\partial p}.$$

In order to obtain $\dot{p}$ from (4), for n=3 it is sufficient to apply the relationship $\dot{p}=J_{K^{-1}}^{-1}\left(p\right)\dot{q}$ because the Jacobian matrix is invertible, whereas for n>3, it is common practice to minimize the 2-norm of the difference of the first and the second term of (4). This leads to an optimal solution ${\dot{p}}_{opt}$, given by:

(6) $${\dot{p}}_{opt}=\min_{\dot{p}}{∥J_{K^{-1}}\left(p\right)\dot{p}-\dot{q}∥}_{2}^{2}=J_{K^{-1}}^{\#}\left(p_{opt}\right)\dot{q},$$

where $J_{K^{-1}}^{\#}\left(p\right)$ is the left pseudo-inverse matrix of $J_{K^{-1}}\left(p\right)$, given by:

(7) $$J_{K^{-1}}^{\#}\left(p\right)={\left(J_{K^{-1}}^{T}\left(p\right)\;J_{K^{-1}}\left(p\right)\right)}^{-1}J_{K^{-1}}^{T}\left(p\right),$$

which exists because the Jacobian matrix $J_{K^{-1}}\left(p\right)$ is full-rank column.

### 2.2. A New Closed-Loop Localization Algorithm

**Figure 1:**
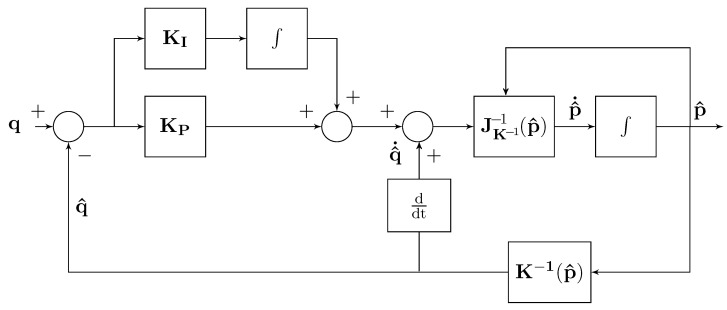
Block scheme of the localization method.

Equation (6) suggests to determine the position vector by means of the following iterative algorithm:

(8) $$\dot{\hat{p}}=J_{K^{-1}}^{\#}\left(\hat{p}\right)\left(\dot{\hat{q}}+K_{P}e+K_{I}\int_{0}^{t}e\left(\tau \right)d\tau \right).$$

where $e=(q-\hat{q})$. The corresponding closed-loop block scheme of the localization method is given in Figure 1.

This algorithm differs from that shown in [32] for the presence of the derivative of the computed feedback distance vector, $\dot{\hat{q}}$, instead of the derivative of the true distances, $\dot{q}$. While the computed feedback distance vector is a continuous function, and consequently numerically derivable, the measured distance vector is a vector whose elements are piecewise constant functions, and consequently cannot be numerically derivable. For this reason, the computation of $\dot{q}$ requires the use of accelerometers, as discussed in [32]. In order to compute a numeric derivative of $\hat{q}$ it is possible to use the robust first-order Levant’s differentiator [35], given by:

(9) $$\begin{array}{c} {\dot{\hat{q}}}_{i}=u_{i}-\lambda \sqrt{\left|(x_{i}-q_{i})\right|}\mathrm{sgn}\left(x_{i}-q_{i}\right), \end{array}$$

(10) $$\begin{array}{c} {\dot{u}}_{i}=-\alpha \mathrm{sgn}\left(x_{i}-q_{i}\right),\\ i=1,\cdots ,n, \end{array}$$

where sgn denotes the signum function.

### 2.3. Using Three Anchors

When the localization is performed using three anchors, it is easy to prove the following theorem.

**Theorem** **1.** *Given a mobile object, let the point P be the center of gravity. Assume that three anchors are employed for the localization of the mobile object, i.e., $q\in R^{3}$. Then (8) is an estimator of the coordinates of the point P, where $J_{K^{-1}}^{\#}\left(\hat{p}\right)=J_{K^{-1}}^{-1}\left(\hat{p}\right)$$e\in R^{3}$ and $K_{P},K_{I}\in R^{3\times 3}$, with $K_{P}=k_{P}I_{3}$ and $K_{I}=k_{I}I_{3}$, provided that $\frac{k_{I}}{k_{P}}>0$.*

*Proof*. Since $\dot{\hat{q}}=J_{K^{-1}}\left(\dot{\hat{p}}\right)$ (cf. (4)), (8) becomes:

(11) $$J_{K^{-1}}^{-1}\left(\hat{p}\right)\left(K_{P}e+K_{I}\int_{0}^{t}e\left(\tau \right)d\tau \right)=0.$$

since $J_{K^{-1}}^{-1}\left(\hat{p}\right)$ is a square full-rank matrix, (11) implies that:

(12) $$k_{P}e+k_{I}\int_{0}^{t}e\left(\tau \right)d\tau =0.$$

It follows that e(t) converges asymptotically to zero when *t* diverges to infinity, provided that $\frac{k_{I}}{k_{P}}>0$. This implies that $\hat{q}$ converges to q and, consequently, $\hat{p}=K\left(\hat{q}\right)$ converges to p=K(q).

### 2.4. Using n>3 Anchors

When n>3 anchors are employed for the localization task, the left pseudo-inverse $J_{K^{-1}}^{\#}\left(\hat{p}\right)$ is a 3×n matrix having rank three. In this case (8) does not imply the convergence of e(t) to zero; in fact, since the *n* columns of $J_{K^{-1}}^{\#}\left(\hat{p}\right)$ are linearly dependent, Equation (8) can be also satisfied for $e_{i}$, i=1,…,n different from zero. However, since the elements of the the Jacobian matrix $J_{K^{-1}}\left(\hat{p}\right)$ are bounded, the elements of $J_{K^{-1}}^{\#}\left(\hat{p}\right)$ are bounded as well, and, consequently, ${∥e∥}_{2}$ is bounded. This implies that ${∥K^{-1}\left(\hat{p}\right)-K^{-1}\left(p\right)∥}_{2}$ is bounded and, since $K^{-1}\left(p\right)$ is a continuous function in $R^{3}$, $∥\hat{p}{-p∥}_{2}$ is bounded.

### 2.5. Experimental Results Using Four Anchors

For the localization experimental tests, the setup described in the Appendix A was used. In this case, where only position estimation was addressed (no speed estimation), the results were compared with the previous results obtained with the algorithm proposed in [32]. In order to highlight the differences between the two estimators, the tests were named as shown in Table 1.

**Figure 2:**
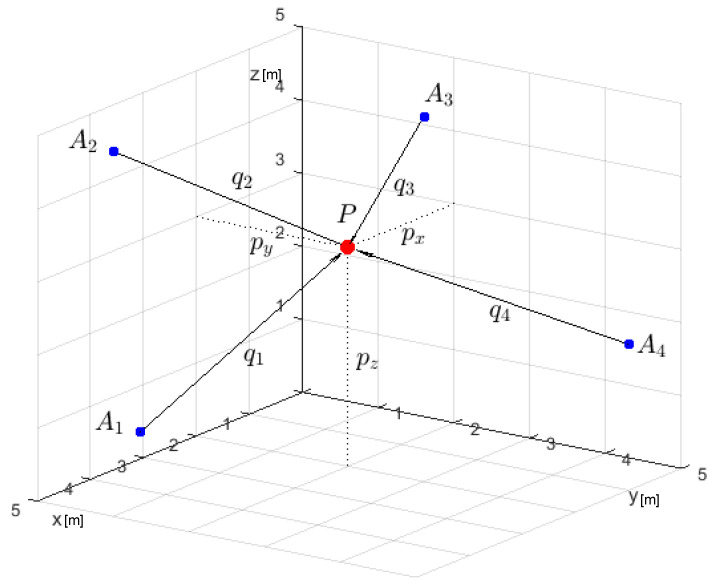
Placement of the anchors in the reference frame.

In particular, the reference motion of the point *P*, corresponding to the onboard mobile anchor, is that depicted in Figure 2. This motion occurs in a scenario in which four fixed anchors are placed in positions $A_{1}=\left(4.58,4.58,1.60\right)$, $A_{2}=\left(4.58,0,2.20\right)$, $A_{3}=\left(0,0,1\right)$, and $A_{4}=\left(0,4.58,2.80\right)$, referred to a conventional Cartesian reference frame. The distances $q_{i},i=1,\ldots ,4$ are measured at a frequency rate of 12 Hz from the onboard mobile anchor and these four fixed anchors $A_{i},i=1,\ldots ,4$.

These measured distances represent the components of the distances vector q applied as input to the closed loop localization scheme of Figure 1. In this localization scheme, the measured distances q are staircase waveforms, whereas the feedback distances $\hat{q}$ are continuous, and then differentiable waveforms, which track, on average, the input distance vector components.

**Figure 3:**
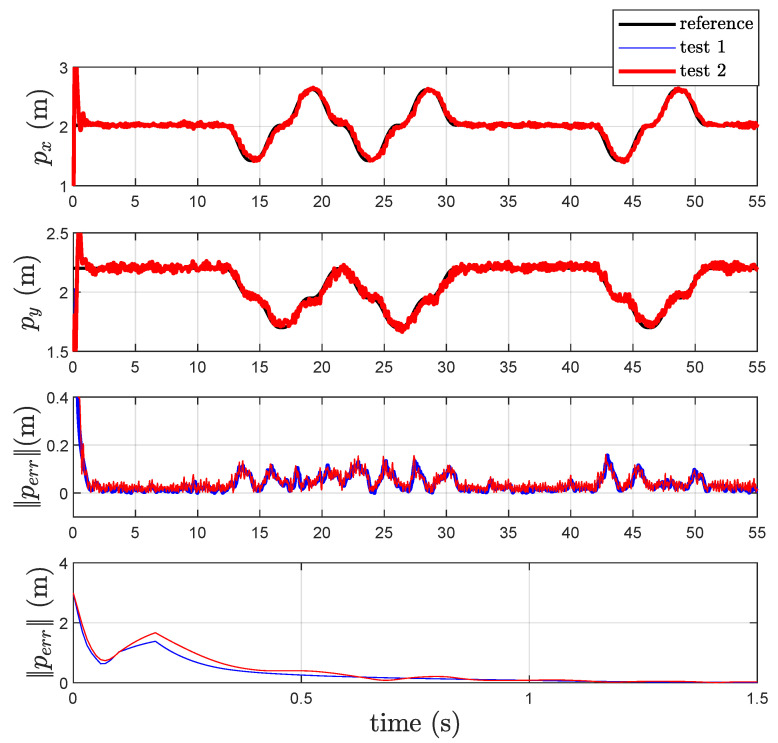
References, estimated coordinates of *P*, and corresponding norm-2 error $∥P_{err}∥$ in *Test 1* and *Test 2*.

**Figure 4:**
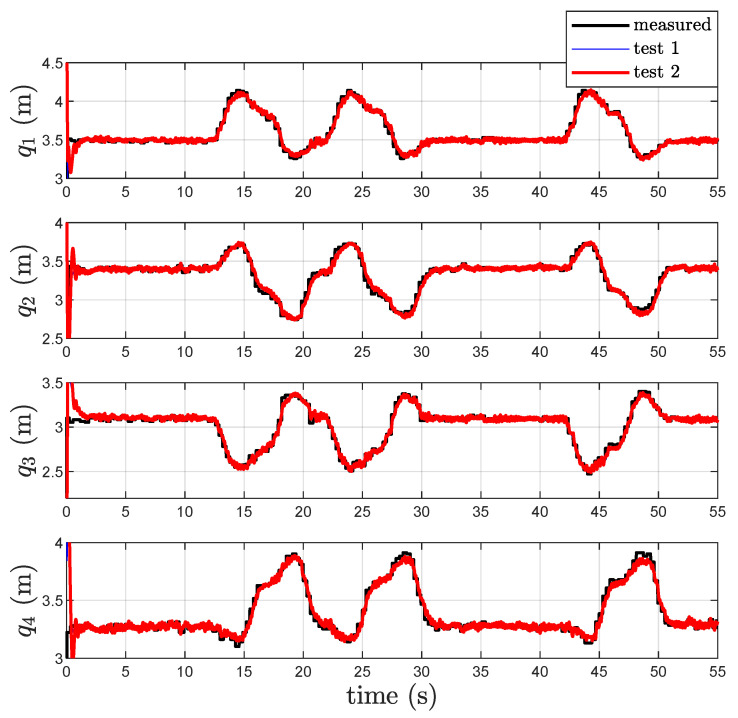
Measured and estimated distances $q_{i}$ in *Test 1* and *Test 2*.

The parameters of the localization scheme are $k_{P}=10$ and $k_{I}=25$. The components of the closed-loop localization scheme output vector, i.e., the coordinates of the point *P*, are given in Figure 3 together with the components of the reference trajectory of *P* during the motion. In the same figure, the corresponding tracking errors for both *Test 1* and *Test 2* are given together with a zoom relative to the initial transient. For both *Test 1* and *Test 2*, the measured distances are reported in Figure 4 in black color. Examination of these figures shows that the new closed loop localization scheme is able to estimate the components of the point *P* with an error that, after a transient, presents a maximum value of 15 cm. The estimated position error for the new algorithm *Test 2*, obtained without the use of acceleration measurement, appears to be a little bit noisier than the one obtained with the previous localization algorithm *Test 1*. The estimated distances $\hat{q}$ for both tests are given in Figure 4. Note that, from Figure 4, it is evident that the two algorithms produce almost the same output and for this reason there is an overlap between the two waveforms (*Test 1* and *Test 2*).

In Table 2, the mean value and the variance of the norm of the position estimation error for both *Test 1* and *Test 2* are reported. Data in Table 1 highlight that mean and variance of the errors relative to *Test 2* are greater than those in *Test 1*. This confirms that using the differentiator instead of the acceleration measurements leads to a small loss of performance which, in turn, is insignificant with respect to the advantage connected to the elimination of the accelerometer itself.

**Remark** **1.** *Note that when the accelerometer is removed from the observer scheme, the algorithm workload is a little bit higher because of the additional computational power needed to implement the differentiator. However, the removal of the accelerometer gives some advantages. In fact, even if accelerometers are very common and not expensive, they are generally affected by systematic errors given by imprecise scaling factors, axes misalignments, non-uniform cross-axis sensitivities, non-zero biases, and temperature dependence that decrease accuracy in the measurements. For these reasons, a system that makes use of accelerometers needs to be initialized with a calibration procedure before use.*

## 3. Velocity Estimation

In order to obtain information about the velocity of *P*, a Kalman–Bucy filter is designed with the aim of obtaining the state estimation of the model, i.e., the position and velocity of *P*.

The strap-down model is given by:

(13) $$\dot{p}=v,$$

(14) $$\dot{v}=a,$$

(15) y=p

where a is the acceleration vector, p and v are the position and velocity vectors, and y is the output vector. The state space model corresponding to (13)–(15) is given by:

(16) $$\dot{x}=Ax+Ba,$$

(17) y=Cx.

where $x={\left[p^{T}v^{T}\right]}^{T}$ is the state vector, and the matrices appearing in (16)–(17) are given by:

$$A=\left[\begin{array}{cc} 0_{3} & I_{3}\\ 0_{3} & 0_{3} \end{array}\right],B=\left[\begin{array}{c} 0_{3}\\ I_{3} \end{array}\right],C=\left[\begin{array}{cc} I_{3} & 0_{3} \end{array}\right],$$

where $0_{3}$ and $I_{3}$ are the null and identity 3×3 matrices. It easy to verify that model (16)–(17) is observable and, consequently, it is possible to employ an observer to obtain the state variables. Due to the linearity of the model (16)–(17) and taking into account that the acceleration, measured by the accelerometers, is affected by a large amount of noise, it is convenient to design a continuous-time Kalman–Bucy filter (KBF) estimator. Moreover, a particle filter is considered for comparison reasons.

**Figure 5:**
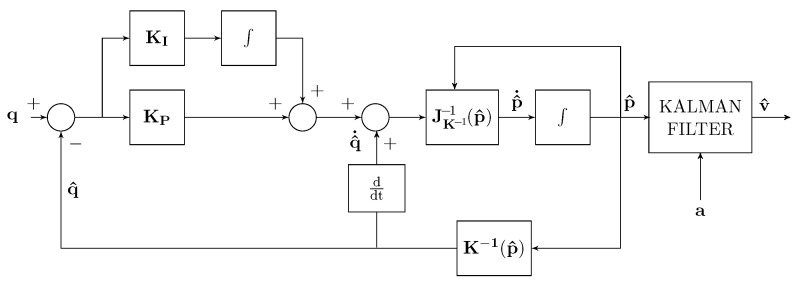
*Test 3* estimator scheme with Kalman filter and differentiator.

**Figure 6:**
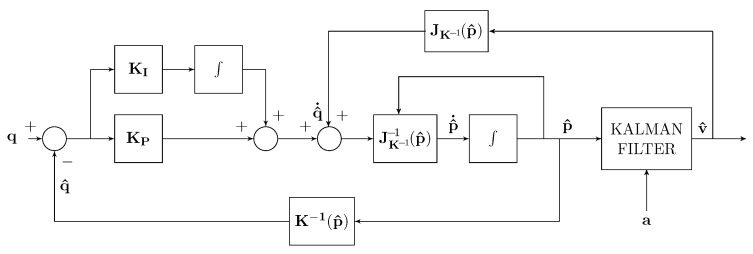
*Test 4* estimator scheme with Kalman filter and velocity feedback.

**Figure 7:**
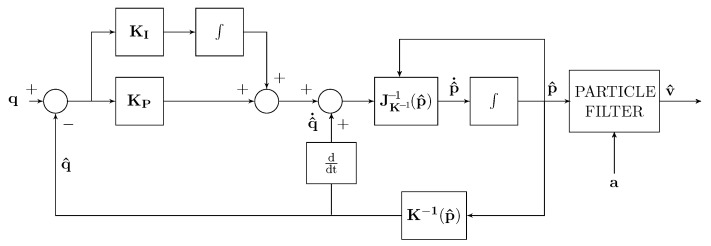
*Test 5* estimator scheme with particle filter and differentiator.

With regard to the velocity estimation, three different implementation schemes were considered, as reported in Table 3. In all the three schemes, it is assumed that the position estimation is that given by the localization scheme of Figure 1. In particular, in Figure 5, a Kalman–Bucy filter is added to the localization scheme of Figure 1 with the aim of obtaining the velocity estimation. In Figure 6, the estimated velocity is used for estimating $\dot{\hat{q}}$ without the use of the differentiator. In Figure 7, a particle filter is used instead of the Kalman–Bucy filter together with the differentiator.

### 3.1. Kalman–Bucy Filter

In order to design the KBF corresponding to the acceleration model, it is necessary to associate with the deterministic model (16)–(17) a stochastic model which takes into account the noise generated by the accelerometer $w_{a}$ together with the true acceleration $\bar{a}$ and the measurement noise $w_{p}$ at the output of the localization scheme $\hat{p}$. Then, the stochastic model of the accelerometer is given by:

(18) $$\dot{x}=Ax+B\bar{a}+Bw_{a},$$

(19) $$y=Cx+w_{p},$$

where $w_{a}$ and $w_{p}$ are uncorrelated Gaussian white noises, $\bar{a}$ is the true acceleration, and y is the output, having, respectively, covariance matrices Q and R. The optimal KBF estimator is therefore given by:

(20) $$\dot{\hat{x}}=A\hat{x}+B\bar{a}+K_{F}\left(t\right)\left[y-C\hat{x}\right],$$

where $K_{F}\left(t\right)=P\left(t\right)C^{T}R^{-1}$ and P(t) is the covariance matrix of the estimation error given by $P\left(t\right)=E\{\left[x\left(t\right)-\hat{x}\left(t\right)\right]{\left[x\left(t\right)-\hat{x}\left(t\right)\right]}^{T}\}$, which is the solution of the following Riccati equation:

(21) $$\dot{P}\left(t\right)=AP\left(t\right)+P\left(t\right)A^{T}+BQB^{T}-K_{F}\left(t\right)RK_{F}^{T}\left(t\right),$$

corresponding to $P\left(0\right)=E\{\left[x\left(0\right)-\hat{x}\left(0\right)\right]{\left[x\left(0\right)-\hat{x}\left(0\right)\right]}^{T}\}$.

**Figure 8:**
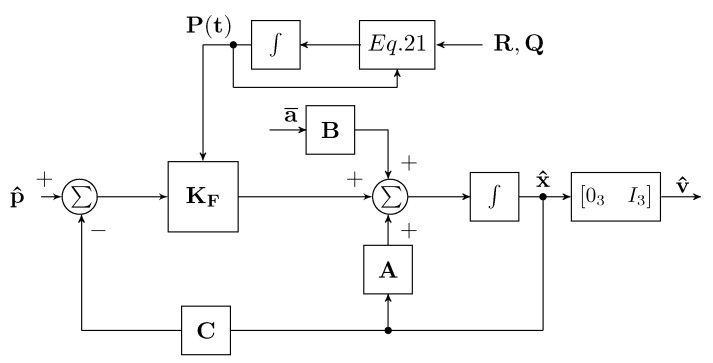
Kalman–Bucy filter block scheme.

To better clarify how the Kalman–Bucy filter is implemented for the velocity estimation, in Figure 8, the corresponding block diagram is reported.

In this way, the localization is performed without using the accelerometers, whereas the velocity estimation requires to employ the signals generated by the accelerometers and a Kalman–Bucy filter. Now, it is interesting to evaluate the use of the estimated velocity for determining the feedback variable $\dot{\hat{q}}$, with the aim of eliminating the differentiator.

Observing (4), it is possible to obtain an estimation of $\dot{\hat{q}}$ starting from the estimation of $\hat{v}$ given by the Kalman–Bucy filter. Obviously, this approach should imply the use of the accelerometer signals also for the localization of the point *P*. It is assumed to again use the position estimated by means of the localization system for obtaining the feedback distance vector $\hat{q}$.

### 3.2. Particle Filter as Alternative to the Kalman–Bucy Filter

In this subsection, the opportunity of using a particle filter instead of a Kalman–Bucy filter is evaluated for velocity estimation of the point *P*. The particle filter is tuned according to the discrete-time model associated with the strap-down model given by (13) and (14). This model is obtained by integrating Equation (14) and, successively, Equation (13) from $t_{0}$ to *t*, starting from the initial conditions $p(t_{0})$ and $v(t_{0})$, and assuming $p\left(t\right)=p\left(kT_{s}\right)$, $v\left(t\right)=v\left(kT_{s}\right)$ and $a_{k}=a\left(kT_{s}\right)$ for $t\in [kT_{s},\left(k+1\right)T_{s})$. Then, the discrete-time model is obtained, placing $t_{0}=kT_{s}$ and $t=\left(k+1\right)T_{s}$ where k=1,2,…. The model in question is represented as follows [24]:

(22) $$x_{k+1}=A_{d}x_{k}+B_{d}{\bar{a}}_{k}+B_{d}w_{a,k},$$

(23) $$y_{k}=Cx_{k}+w_{p,k},$$

where $a_{k}={\bar{a}}_{k}+w_{a,k}$, with ${\bar{a}}_{k}$ the true acceleration and $w_{a,k}$ the superimposed accelerometer noise, and

(24) $$A_{d}=\left[\begin{array}{cc} I_{3} & T_{s}I_{3}\\ 0_{3} & I_{3} \end{array}\right];B_{d}=\left[\begin{array}{c} 0.5T_{s}^{2}I_{3}\\ T_{s}I_{3} \end{array}\right]$$

Placing $\epsilon =B_{d}w_{a,k}$, the covariance matrix $Q_{\epsilon }=E\left\{\epsilon \epsilon^{T}\right\}$ is given by:

(25) $$Q_{\epsilon }=\left[\begin{array}{cc} \frac{1}{4}T_{s}^{4} & \frac{1}{2}T_{s}^{3}\\ \frac{1}{2}T_{s}^{3} & T_{s}^{2} \end{array}\right]⊗\Sigma_{3},$$

where $\Sigma_{3}=diag\left(\sigma_{x}^{2},\sigma_{y}^{2},\sigma_{z}^{2}\right)$ and $\sigma_{x}$, $\sigma_{y}$, and $\sigma_{z}$ are standard deviations of the accelerometer noise along the axes (*x*, *y*, and *z*).

The particle filter considered here is shown in [36] (the one available in the MATLAB environment). It requires the deterministic discrete-time plant model (22)–(23) without noises $w_{a,k}$ and $w_{p,k}$, and with $a_{k}$ instead of ${\bar{a}}_{k}$, and the construction of two functions, the state transition and the likelihood function.

The state transition function computes a 6×Np matrix where Np is the particles number, and the *j*th column is the state value corresponding to the *j*th particle. The likelihood function computes the likelihood of each particle, starting from the output error given by the difference of the measured output and the output computed with the state associated to the particle itself. The output is then computed starting from the state of the particle having the maximum likelihood.

### 3.3. Experimental Results for the Velocity Estimation Schemes

To properly design the KBF it is necessary to obtain the covariance matrices Q and R. To determine Q, the following procedure is performed. The three components of the acceleration vector, referred to in the conventional frame of Figure 2 and acquired during the experiment described in the previous section, are passed offline through three anticausal filters in order to split each acceleration component into the true acceleration and the noise signal superimposed to them. The anticausal filters are based on a continuous-time third-order Butterworth filter, having different bandwidths so that the mean value of the three noise signals is almost the same and is as low as possible. Then, the three matrix elements of Q, chosen diagonal, are computed as the covariances of the above noise signals. The covariance matrix, computed assuming the three elements of Q equal to the maximum value of the covariance along the three axes, is given by Q=diag(0.2244,0.2244,0.2244). The covariance matrix R is chosen equal to $I_{3}$.

To design the particle filter, the distribution function is assumed Gaussian with a null mean and covariance ${\left[\begin{array}{cccccc} 0.0001 & 0.0001 & 0.0001 & 0.2244 & 0.2244 & 0.2244 \end{array}\right]}^{T}$, and the particle number is assumed to be $N_{p}=10000$. The first three components of the covariance vector represent the covariances of the position component of *P*, whereas the remaining three components represent the covariances of the three acceleration components. The sample time is that corresponding to the frequency of 100 Hz, as in the previous experimental results.

**Figure 9:**
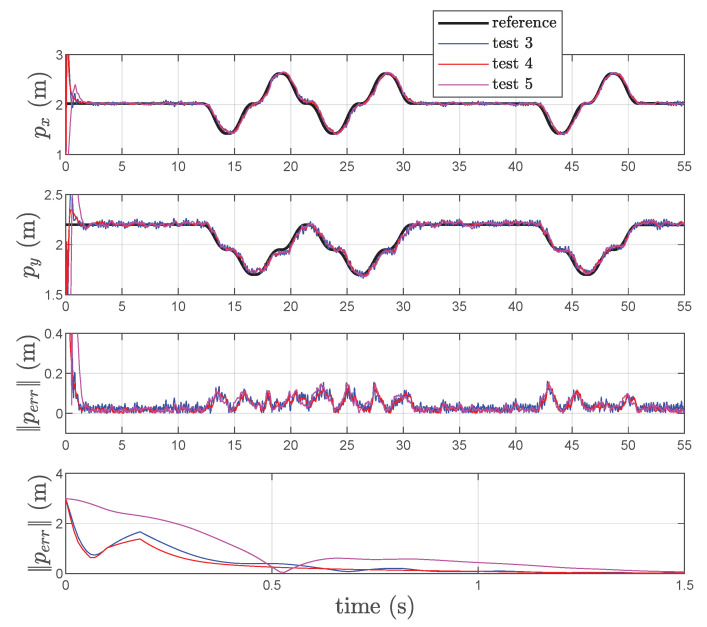
References, estimated coordinates of *P*, and corresponding norm-2 error $∥P_{err}∥$ in *Test 3, 4* and *Test 5*.

**Figure 10:**
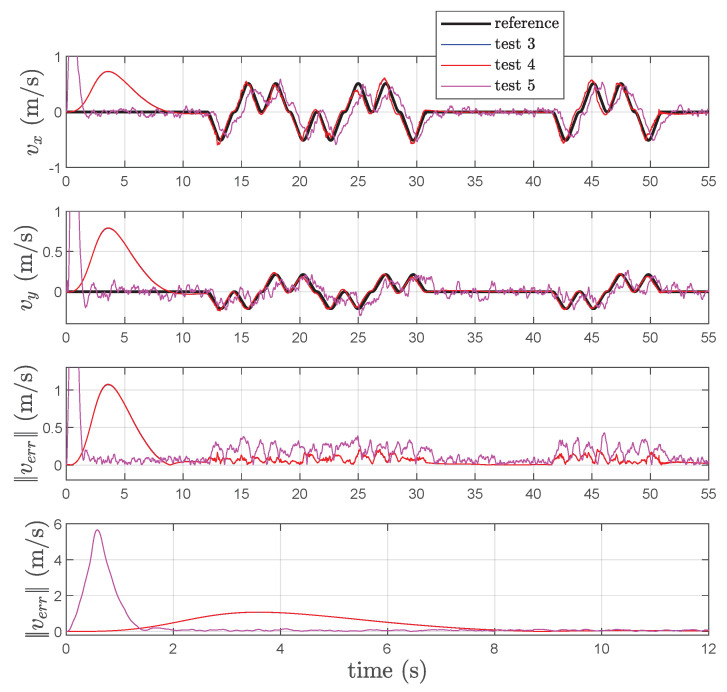
References, estimated velocities $\hat{v}$, and corresponding norm-2 error $∥v_{err}∥$ in *Test 3, 4* and *Test 5*.

In Figure 9, the estimated position, relative to *Test 3, 4* and *5*, is reported while the experimental results relative to the velocity estimation are given in Figure 10. Examination of this figure shows that the KBF is able to give an acceptable estimation of the velocity of point *P* in both cases of presence of the differentiator (cf. Figure 5), and direct feedback of the estimated velocity (cf. Figure 6). Instead, the particle filter is not able to estimate the velocity of *P* or considerably increase the number of particles. Examination of this figure shows that the effects of the velocity feedback on the localization are those of introducing noise in the estimated position of *P* together with a variation of the mean value of the estimation error and its variance, as is confirmed by Table 4. On the other end, even if the particle filter is able to reproduce the position with an acceptable mean error (greater than the one obtained using the KBF), the variance is four times higher.

**Figure 11:**
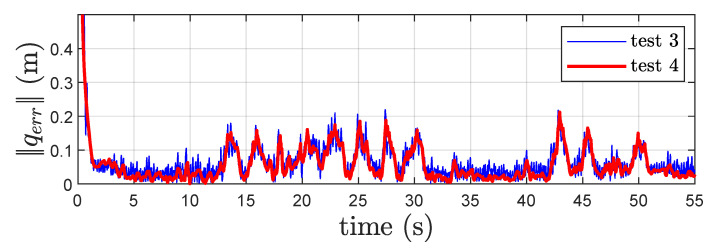
Norm of the distance estimation error $∥q_{err}∥$ for *Test 3* and *Test 4*.

Finally, in Figure 11, the norm of the distance estimation error is shown for *Test 3* and *4*. It appears that results from *Test 4* present a better performance in terms of mean value and variance with respect to the one in *Test 3*.

In conclusion, from the experimental tests, it is possible to affirm that the best results are obtained using the differentiator and the Kalman–Bucy filter for velocity estimation, as shown in Figure 5, *Test 3*. In fact, this structure allows to obtain, especially in the position estimation task, a lower mean and variance for the position estimation error (see Table 4) while it behaves in a similar way to *Test 4* and *Test 5* for the velocity estimation (see Table 5). Alternatively, it is possible to use the Kalman–Bucy filter alone, and feed back the velocity estimated by the filter, avoiding the use of the differentiator, accepting an increasing of the mean value and the variance of the position estimation error. The scheme with the particle filter, *Test 5*, is instead to be excluded because in both cases (position and speed estimation) it behaves worse than the other two solutions proposed in *Test 3* and *Test 4*.

## 4. Conclusions

In this work, a novel observer for a range-based localization system is proposed for applications in environments where the GPS fails. Two aspects were developed. Firstly, a closed-loop localization scheme, which uses the measured distances as input variable, was designed. Secondly, a Kalman–Bucy filter was used to estimate the velocity components of *P*, and it was compared with the particle filter. Experimental results show the effectiveness of the proposed localization scheme. In particular, it was shown that it is possible to approximate the first derivatives of the distances by using a suitable developed differentiator. This leads to a slightly noisier estimate, but allows to eliminate the accelerometer and all problems connected to it. Moreover, the Kalman–Bucy filter results as the best-performing filter for estimating the velocity of point *P*, compared with the particle filter. Finally, it was shown that the velocity estimated by the Kalman–Bucy filter can be fed back to estimate the derivative of the distance vector, avoiding the use of a differentiator to implement the proposed localization algorithm.

## Figures and Tables

**Table 1:** Experimental tests for the localization without speed estimation.

Test Name	Estimation Algorithm
*Test 1*	The one already proposed in [32].
*Test 2*	Localization without accelerometers as in Equation (8).

**Table 2:** Mean value and variance of the position estimation error in *Test 1* and *Test 2*.

	Mean Value [m]	Variance [m${}^{2}$]
*Test 1*	0.045	0.00910
*Test 2*	0.053	0.01187

**Table 3:** Experimental tests for the localization with speed estimation.

Test Name	Estimation Algorithm
*Test 3*	Differentiator and Kalman–Bucy filter (cf. Figure 5).
*Test 4*	Estimation of $\dot{\hat{q}}$ by Kalman–Bucy filter (cf. Figure 6).
*Test 5*	Differentiator and particle filter (cf. Figure 7).

**Table 4:** Mean value and variance of the position estimation error in *Test 3*, *Test 4*, and *Test 5*.

	Error Mean Value [m]	Error Variance [m${}^{2}$]
*Test 3*	0.045	0.00911
*Test 4*	0.053	0.01187
*Test 5*	0.064	0.04008

**Table 5:** Mean value and variance of the speed estimation error in *Test 3*, *Test 4*, and *Test 5*.

	Error Mean Value [m]	Error Variance [m${}^{2}$]
*Test 3*	0.125	0.04860
*Test 4*	0.125	0.04877
*Test 5*	0.198	0.20147

**Figure A1:**
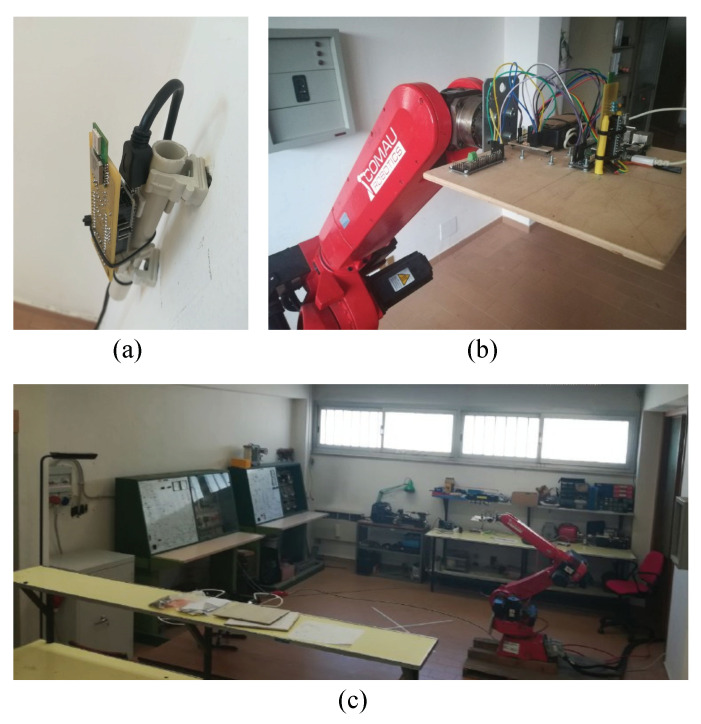
Photo of the experimental setup: (**a**) fixed beacon, (**b**) mobile plate to be tracked, (**c**) indoor scenario.

## Data Availability

Not applicable.

## Appendix A. Description of the Experimental Setup

The experimental tests were conducted by bringing the equipment on a plate mounted on the end effector of a Smart Six-1.4 industrial robot Comau that serves as ground-truth reference. The plate is equipped with a 9-axis IMU LSM9DSI from STMicroelectronics (used only for *Tests 3, 4*, and *5* defined later in the paper, where the velocity estimation was also performed), a Raspberry Pi3 SBC, which runs the compiled code generated by means of Matlab/Simulink environment at a frequency of 100 Hz, and an anchor device built on top of a Decawave DWM1000 radio transceiver. The robot motion is controlled by means of the Comau C4G controller, which is also able to exchange data with the Raspberry SBC via a WiFi connection. In this way, it is possible to impose the highly accurate and a priori known desired motion to the plate, and to have accurate trajectory references for both point *P* and the distances between the onboard anchor and the fixed ones.

Figure A1 shows a photo of the experimental setup. The UWB ranging system is composed of four fixed devices placed on the scene and a mobile device placed on the object to be tracked. The beacon hardware is based on DW1000 IC from Decawave, a single-chip IEEE802.15.4-2011UWB compliant device with internal high-precision counter (each LSB of the timestamp timer is 15.65 pS, which leads to maximum theoretical accuracy of ±5 mm) for time-of-flight (ToF) measurement.

Since a two-way ranging technique was used to perform ToF measurements, more time captures are needed to compute a single distance measurement. This led to a measurement accuracy, as indicated on the DW1000 manufacturer website, of ±10 cm instead of the maximum theoretical accuracy of ±5 mm. Moreover, each beacon is equipped with an external microcontroller. The range measurement system has an actual equivalent refresh rate of up to 12 Hz.

The experimental implementation was carried out by means of Matlab/Simulink. In particular, the Raspberry runs a precompiled C code built from the Simulink scheme of the proposed algorithm. Real-time execution, in external mode, has a frequency of 100 Hz. UWB and IMU devices are connected to the Raspberry by mean of I2C digital Bus. I2C and LSM9DS1 Simulink blocks are used for interfacing the sensor measurements with the Simulink scheme of the controller.

## References

[1] Monica S., Ferrari G. (2020). Robust UWB-Based Localization with Application to Automated Guided Vehicles. Adv. Intell. Syst..

[2] Shakhatreh H., Sawalmeh A.H., Al-Fuqaha A., Dou Z., Almaita E., Khalil I., Othman N.S., Khreishah A., Guizani M. (2019). Unmanned Aerial Vehicles (UAVs): A Survey on Civil Applications and Key Research Challenges. IEEE Access.

[3] Alonge F., D’Ippolito F., Fagiolini A., Garraffa G., Sferlazza A. (2021). Trajectory robust control of autonomous quadcopters based on model decoupling and disturbance estimation. Int. J. Adv. Robot. Syst..

[4] Hamel T., Mahony R. Attitude estimation on SO(3) based on direct inertial measurements. Proceedings of the IEEE International Conference on Robotics and Automation.

[5] Fossen T.I. (2011). Handbook of Marine Craft Hydrodynamics and Motion Control.

[6] Zhang W., Ghogho M., Yuan B. (2012). Mathematical Model and Matlab Simulation of Strapdown inertial navigation system. Model. Simul. Eng..

[7] Fossen T.I. (1994). Guidance and Control of Ocean Vehicles.

[8] Fossen T.I. (2002). Marine Control Systems: Guidance, Navigation and Control of Ships, Rigs and Underwater Vehicles.

[9] Paull L., Saeedi S., Seto M., Li H. (2014). AUV navigation and localization: A review. Ocean. Eng. IEEE J..

[10] Alonge F., D’Ippolito F., Garraffa G., Sferlazza A. (2019). A Hybrid Observer for Localization of Mobile Vehicles with Asynchronous Measurements. Asian J. Control.

[11] Li M.G., Zhu H., You S.Z., Tang C.Q. (2020). UWB-Based Localization System Aided With Inertial Sensor for Underground Coal Mine Applications. IEEE Sens. J..

[12] Lee J., Moon J., Kim S. UWB-based Multiple UAV Control System for Indoor Ground Vehicle Tracking. Proceedings of the 2021 IEEE VTS 17th Asia Pacific Wireless Communications Symposium (APWCS).

[13] Lee H., Kim W., Seo J. Simulation of UWB Radar-Based Positioning Performance for a UAV in an Urban Area. Proceedings of the 2018 IEEE International Conference on Consumer Electronics-Asia (ICCE-Asia).

[14] Zafari F., Gkelias A., Leung K.K. (2019). A Survey of Indoor Localization Systems and Technologies. IEEE Commun. Surv. Tutor..

[15] Yassin A., Nasser Y., Awad M., Al-Dubai A., Liu R., Yuen C., Raulefs R., Aboutanios E. (2017). Recent Advances in Indoor Localization: A Survey on Theoretical Approaches and Applications. IEEE Commun. Surv. Tutor..

[16] Alarifi A., Al-Salman A., Alsaleh M., Alnafessah A., Al-Hadhrami S., Al-Ammar M.A., Al-Khalifa H.S. (2016). Ultra wideband indoor positioning technologies: Analysis and recent advances. Sensors.

[17] Dardari D., Closas P., Djurić P.M. (2015). Indoor Tracking: Theory, Methods, and Technologies. IEEE Trans. Veh. Technol..

[18] You W., Li F., Liao L., Huang M. (2020). Data Fusion of UWB and IMU Based on Unscented Kalman Filter for Indoor Localization of Quadrotor UAV. IEEE Access.

[19] Yao L., Wu Y.W.A., Yao L., Liao Z.Z. An integrated IMU and UWB sensor based indoor positioning system. Proceedings of the 2017 International Conference on Indoor Positioning and Indoor Navigation (IPIN).

[20] Kulikov R.S. Integrated UWB/IMU system for high rate indoor navigation with cm-level accuracy. Proceedings of the 2018 Moscow Workshop on Electronic and Networking Technologies (MWENT).

[21] Foy W.H. (1976). Position-location solutions by Taylor-series estimation. IEEE Trans. Aerosp. Electron. Syst..

[22] Manolakis D.E. (1996). Efficient solution and performance analysis of 3-D position estimation by trilateration. IEEE Trans. Aerosp. Electron. Syst..

[23] Navidi W., Murphy W.S., Hereman W. (1998). Statistical methods in surveying by trilateration. Comput. Stat. Data Anal..

[24] Guo K., Qiu Z., Miao C., Zaini A.H., Chen C.L., Meng W., Xie L. (2016). Ultra-wideband-based localization for quadcopter navigation. Unmanned Syst..

[25] Murphy W.S., Hereman W. (1995). Determination of a Position in Three Dimensions Using Trilateration and Approximate Distances.

[26] Coope I. (2000). Reliable computation of the points of intersection of n spheres in Rn. ANZIAM J..

[27] Cao M., Anderson B.D., Morse A.S. Localization with imprecise distance information in sensor networks. Proceedings of the Proceedings of the 44th IEEE Conference on Decision and Control, IEEE, Seville, Spain, 15–15 December 2005.

[28] Thomas F., Ros L. (2005). Revisiting trilateration for robot localization. IEEE Trans. Robot..

[29] Liu X., Zhou B., Huang P., Xue W., Li Q., Zhu J., Qiu L. (2021). Kalman filter-based data fusion of wi-fi rtt and pdr for indoor localization. IEEE Sens. J..

[30] Shaukat N., Ali A., Javed Iqbal M., Moinuddin M., Otero P. (2021). Multi-sensor fusion for underwater vehicle localization by augmentation of rbf neural network and error-state kalman filter. Sensors.

[31] Liu J., Guo G. (2021). Vehicle localization during GPS outages with extended Kalman filter and deep learning. IEEE Trans. Instrum. Meas..

[32] Garraffa G., Sferlazza A., D’Ippolito F., Alonge F. (2022). Localization Based on Parallel Robots Kinematics As an Alternative to Trilateration. IEEE Trans. Ind. Electron..

[33] DecaWave (2017). DW1000 User Manual.

[34] Siciliano B. (1999). The Tricept robot: Inverse kinematics, manipulability analysis and closed-loop direct kinematics algorithm. Robotica.

[35] Levant A. (1998). Robust exact differentiation via sliding mode technique. Automatica.

[36] Chen Z. (2003). Bayesian Filtering: From Kalman Filters to Particle Filters, and Beyond. Statistics.

